# Automated Shoulder Girdle Rigidity Assessment in Parkinson’s Disease via an Integrated Model- and Data-Driven Approach

**DOI:** 10.3390/s25196019

**Published:** 2025-10-01

**Authors:** Fatemeh Khosrobeygi, Zahra Abouhadi, Ailar Mahdizadeh, Ahmad Ashoori, Negin Niksirat, Maryam S. Mirian, Martin J. McKeown

**Affiliations:** 1 School of Kinesiology, University of British Columbia, Vancouver, BC V6T 1Z4, Canada; fatemeh.khosrobeygi@ubc.ca; 2 Control and Intelligent Processing Center of Excellence, School of Electrical and Computer Engineering, College of Engineering, University of Tehran, Tehran 14155-6619, Iran; zahra.abouhadi@ut.ac.ir; 3 Department of Electrical and Computer Engineering, University of British Columbia, Vancouver, BC V6T 1Z4, Canada; ailar.mahdizadeh@ubc.ca; 4 Pacific Parkinson’s Research Centre, Djavad Mowafaghian Centre for Brain Health, University of British Columbia, Vancouver, BC V6T 2B5, Canada; aashoori@ece.ubc.ca (A.A.); maryam.mirian@ubc.ca (M.S.M.); 5 Faculty of Medicine, McGill University, Montreal, QC H3A 0G4, Canada; negin.niksirat@mail.mcgill.ca; 6 Faculty of Medicine (Neurology), University of British Columbia, Vancouver, BC V6T 2B5, Canada

**Keywords:** Parkinson’s disease, rigidity assessment, natural frequency, damping ratio, decay rate, LTI model, weak supervision

## Abstract

**Highlights:**

**What are the main findings?**

- A hybrid framework integrating model-driven (damping ratio, decay rate) and data-driven (maximum detail coefficient) features via weak supervision achieved a strong correlation (r = 0.78, p < 0.001) with UPDRS rigidity scores, outperforming traditional Wartenberg pendulum test metrics like maximum velocity.
- The integrated model improved PD/HC classification accuracy by 10% over data-driven methods, with damping ratio and maximum detail coefficient identified as highly predictive biomarkers.

**What is the implication of the main finding?**

- Combining biomechanical and statistical features through weak supervision enables objective, interpretable shoulder rigidity assessment in Parkinson’s Disease. The results suggest that rigidity, generally considered velocity-independent, can be inferred by velocity-dependent features like the damping ratio.
- Because rigidity assessment typically requires in-person, hands-on examination, wearable sensors in the current framework enable scalable, remote monitoring that facilitates earlier diagnosis and ongoing longitudinal tracking within telemedicine settings.

**Abstract:**

Parkinson’s disease (PD) is characterized by motor symptoms, with key diagnostic features, such as rigidity, traditionally assessed through subjective clinical scales. This study proposes a novel hybrid framework integrating model-driven biomechanical features (damping ratio, decay rate) and data-driven statistical features (maximum detail coefficient) from wearable sensor data during a modified pendulum test to quantify shoulder girdle rigidity objectively. Using weak supervision, these features were unified to generate robust labels from limited data, achieving a 10% improvement in PD/healthy control classification accuracy (0.71 vs. 0.64) over data-driven methods and matching model-driven performance (0.70). The damping ratio and decay rate, aligning with Wartenberg pendulum test metrics like relaxation index, revealed velocity-dependent aspects of rigidity, challenging its clinical characterization as velocity-independent. Outputs correlated strongly with UPDRS rigidity scores (*r* = 0.78, *p* < 0.001), validating their clinical utility as novel biomechanical biomarkers. This framework enhances interpretability and scalability, enabling remote, objective rigidity assessment for early diagnosis and telemedicine, advancing PD management through innovative sensor-based neurotechnology.

## 1. Introduction

Parkinson’s disease (PD) is a progressive neurodegenerative disorder affecting over 10 million people worldwide, characterized by a range of motor symptoms including tremor, bradykinesia, postural instability, and rigidity [1]. Among these, rigidity, defined as increased resistance to passive limb movement, is a core diagnostic criterion and a major contributor to disability in PD [2]. Despite its clinical relevance, rigidity is primarily assessed using subjective clinical scales such as the Unified Parkinson’s Disease Rating Scale (UPDRS), where examiners assign scores based on their perception of joint stiffness during passive movement [3]. This approach suffers from significant inter- and intra-rater variability, reducing reliability and sensitivity, especially in early-stage disease or treatment monitoring [3].

Existing efforts to quantify rigidity objectively fall into three main categories. First, biomechanical methods such as the Wartenberg pendulum test offer insight into limb stiffness but heavily rely on visual interpretation, which limits their objectivity [4]. Second, robotic and haptic systems provide precise rigidity measurements through controlled forces, yet they remain expensive and impractical for routine or remote clinical use [5]. Third, wearable sensors and inertial measurement units (IMUs) have successfully quantified other PD symptoms like tremor and bradykinesia. Still, they are less commonly applied to rigidity due to the passive nature of the movement involved [6].

Recent advances in machine learning have introduced promising tools for processing sensor data. Yet, most existing models rely solely on either physical models or statistical learning, limiting their generalizability and interpretability [7]. Furthermore, supervised learning approaches depend heavily on large, high-quality labeled datasets, which are difficult to obtain in clinical contexts where labels are often noisy or sparse. Weak supervision has emerged as a novel solution to this challenge, allowing models to be trained using incomplete or noisy labels derived from heuristics, domain knowledge, or preliminary models [8].

It has been increasingly applied to IMU-derived data in PD to address noisy and sparse clinical labels, with demonstrated effectiveness in gait and tremor analysis [9]. It was shown that weak supervision enables accurate PD-stage classification in natural environments with limited annotation, and similar methodologies have been used for automatic labeling of gait and movement disorders [10]. However, applications of weak supervision to the objective assessment of rigidity remain limited, as most efforts have focused on active movements, leaving the passive assessment domain—such as rigidity during pendulum tests—largely unexplored.

This study proposes a unified framework for objectively quantifying shoulder girdle rigidity in PD by integrating model-driven biomechanical features and data-driven statistical features extracted from wearable sensor data collected during a modified pendulum test. Crucially, these features are combined using a weak supervision strategy that reduces dependence on fully labeled data and enhances interpretability. The primary aim of this study is to develop and validate a hybrid framework that integrates model-driven biomechanical features with data-driven statistical features, unified through weak supervision, to enhance the objectivity, interpretability, and scalability of shoulder girdle rigidity assessment in Parkinson’s Disease, thereby improving its clinical applicability for diagnosis and remote monitoring. Our findings suggest that this hybrid framework can match the accuracy of purely model-driven approaches while offering superior generalizability and more substantial alignment with clinical UPDRS rigidity scores, highlighting its potential for remote monitoring and early diagnosis. This represents a novel application of weak supervision to passive rigidity assessment, extending prior IMU-based approaches beyond active symptoms and addressing a critical gap in the literature.

## 2. Materials and Methods

### 2.1. Participants and Ethical Approval

This study recruited 19 participants, including nine individuals diagnosed with idiopathic Parkinson’s Disease (PD) at mild to moderate severity (Hoehn and Yahr stages 2–3) and 10 age-matched healthy controls (HC). All participants were recruited through the Pacific Parkinson’s Research Centre (PPRC) at the University of British Columbia (UBC). Inclusion criteria for PD participants included a clinical diagnosis of PD, the ability to remain seated without assistance, and the absence of major musculoskeletal or neurological comorbidities.

The sample size of 19 participants (9 PD, 10 HC) was determined based on feasibility for this pilot study, drawing from similar exploratory IMU-based PD research (e.g., [11], with *n* = 12) While small, it provided sufficient data for proof-of-concept evaluation of the hybrid framework, achieving statistically significant correlations (e.g., r = 0.78, *p* < 0.001). Larger cohorts are planned for validation, as noted in the study limitations.

PD participants were assessed in both their medication “OFF” state—defined as at least 12 h after the last dose of L-DOPA—and their “ON” state (45 min post-L-DOPA), though only “OFF” state data were included in this study. All participants provided written informed consent prior to participation. Ethical approval was granted by the UBC Clinical Research Ethics Board (Protocol ID: [H08-01402]).

Rigidity in PD participants was assessed by trained neurologists at the Pacific Parkinson’s Research Centre (PPRC) using the UPDRS Part III (Motor Examination), specifically item 22 (Rigidity), as part of the clinical evaluation protocol described in the study’s data collection [12]. During the assessment, clinicians passively moved the participants’ limbs (neck, left and right upper extremities, and left and right lower extremities) at varying speeds while instructing participants to remain fully relaxed, evaluating resistance to movement independent of velocity, a hallmark of PD rigidity [2]. Rigidity was scored on a 0–4 scale (0 = absent, 4 = severe) for each body part, with scores reflecting the degree of stiffness and the presence of cogwheel rigidity, if applicable. Assessments were conducted in the medication “OFF” state (≥12 h post-L-DOPA) to capture baseline rigidity, consistent with the modified pendulum test protocol (Section 2.2), which isolated passive shoulder girdle movement to minimize volitional influences like bradykinesia. These UPDRS scores, detailed for each limb in Table S2, served as the clinical reference for validating the sensor-based framework’s outputs.

### 2.2. Experimental Protocol and Data Collection

Participants were seated in an armless chair without head or back support. For the pendulum test, the participant’s arm was passively elevated to shoulder height (horizontal position) and released without warning, allowing it to swing freely under the influence of gravity. They were not asked to perform any ancillary movements (e.g., Froment’s maneuver) [13].

#### Wearable Sensors

**Figure 1:**
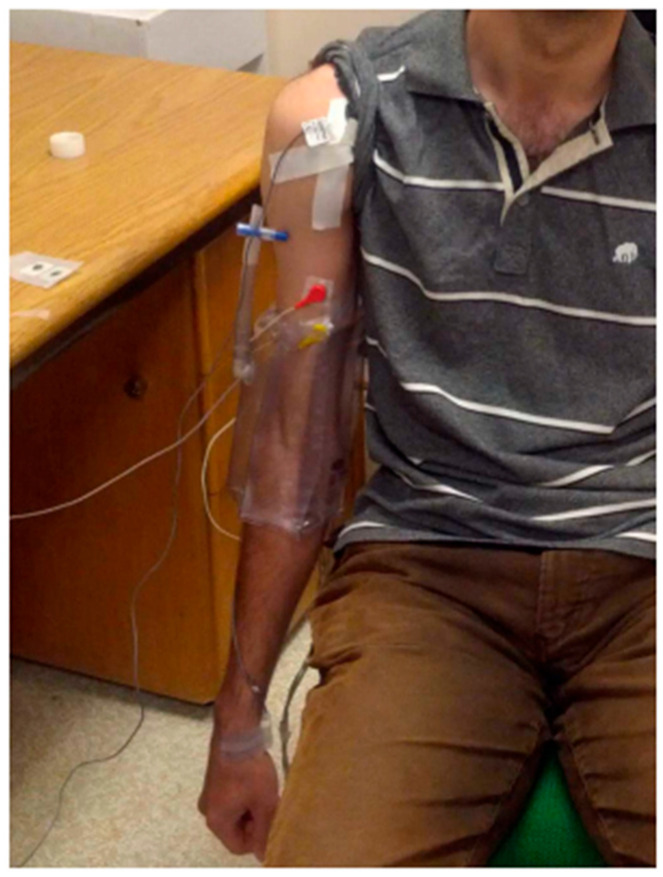
Experimental setup. Subject’s arm was passively moved to the horizontal position. Then, it was released to fall without informing them. They were instructed to relax to the best of their abilities without interfering with the pendulum motion. IMUs were placed on the anterior shoulder and ventrolateral wrist, with the reference system aligned such that the *z*-axis captured primary oscillatory motion (pendulum swing), *x*-axis for lateral deviations, and *y*-axis for vertical components.

As shown in Figure 1, motion data were acquired using lightweight inertial measurement units (IMUs) (Kinetisense Inc., Medicine Hat, AB, Canada), affixed to the anterior surface of the shoulder and the ventrolateral aspect of the wrist using hypoallergenic medical-grade tape. The sensors recorded tri-axial linear acceleration and angular velocity at 128 Hz.

Electromyography (EMG) data were collected using bipolar surface electrodes (Delsys Inc., Natick, MA, USA), placed on the anterior belly of the biceps brachii and lateral head of the triceps brachii. EMG signals were sampled at 2048 Hz to capture muscle activity during passive swing.

Sensor axis alignment and placement procedures followed validated protocols, described in ref. [12]. Further experimental setup details are provided in Section S2 of the Supplementary Materials.

### 2.3. Feature Extraction

Features were extracted from motion signals using a hybrid approach that combines model-driven and data-driven analyses. Preprocessing steps for denoising, alignment, and normalization are described in ref. [12].

#### 2.3.1. Model-Driven Features

Damping Ratio and Decay Rate: Using a second-order linear time-invariant (LTI) model of arm swing dynamics, we estimated the damping ratio and effective decay rate, which serve as proxies for rigidity [12,14]. The damping ratio quantifies the level of damping in the system, while the decay rate reflects the rate at which oscillations diminish. These parameters offer biomechanical insights into the motor function of PD and HC subjects and are directly associated with altered dynamics resulting from rigidity.

Natural Frequency: This parameter, calculated as part of the LTI model analysis, represents the frequency at which the arm would oscillate in the absence of damping [14,15]. *Wn* reflects the intrinsic physical properties of the arm, such as its mass, length, and moment of inertia, but is not influenced by damping or dynamic features. In contrast, the damped natural frequency (*Wd*) incorporates the effects of damping. It is directly linked to dynamic characteristics such as the damping ratio and decay rate, which correlate with rigidity and clinical measures like UPDRS. Together, these parameters provide complementary insights into movement patterns and altered limb dynamics between PD and HC subjects.

#### 2.3.2. Data-Driven Features

Time Domain Features: Statistical descriptors—including area under the curve (AUC), mean, standard deviation, variance, and peak angular velocity—were computed from the angular velocity signals across all three axes to capture the overall amplitude and variability of arm swing, which are indicative of motor abnormalities in PD [14].

Frequency Domain Features: Dominant frequency components were extracted using the Fast Fourier Transform (FFT). Additional frequency features were obtained using wavelet decomposition to capture transient changes. These features capture both sustained rhythmicity and brief disruptions in movement, which are often altered in PD [16].

Time–Frequency Features: Maximum detail and approximation coefficients from continuous wavelet transforms were used to capture combined temporal and spectral patterns [17].

Axis Strategy: Although the z-axis was expected to capture the primary oscillatory motion, as it was along with the pendulum-like oscillation, we extracted features from all three axes (*x*, *y*, and *z*) to assess the potential importance of off-axis movements. Singular value decomposition (SVD) was applied to reorient the data based on the principal axis of motion, further improving feature robustness.

For the complete list of features, the reader might refer to Section S1 of the Supplementary Materials.

### 2.4. Weak Supervision and Label Generation

Snorkel was used to employ a weak supervision approach to label the extracted features without relying on fully annotated datasets. The labeling functions were designed using the mean values of features j derived from PD subjects in their ‘OFF’ state as thresholds (denoted as ${µ}_{j}^{PD}$).

For each feature j, the mean value of the corresponding feature in healthy controls, ${µ}_{j}^{HC}$, was compared to the threshold to guide labeling as follows in (1) and (2):

(1) $$\mathrm{If}\;{µ}_{j}^{HC}>{µ}_{j}^{PD}:\;\;\;\;\;\;\;\;\;\;\;\;\;\;\;L_{ij}=\;\left\{\begin{array}{c} 1\;\;\;\;\;\;\;if\;\;\;F_{ij}<\;{µ}_{j}^{PD}\\ 0\;\;\;\;\;\;\;\;if\;\;F_{ij}\;\geq \;{µ}_{j}^{PD} \end{array}\right.$$

(2) $$\mathrm{If}\;{µ}_{j}^{HC}\leq {µ}_{j}^{PD}:\;\;\;\;\;\;\;\;\;\;\;\;\;\;\;L_{ij}=\left\{\begin{array}{c} 1\;\;\;\;\;\;\;if\;\;\;F_{ij}\geq \;{µ}_{j}^{PD}\\ 0\;\;\;\;\;\;\;if\;\;\;F_{ij}<{µ}_{j}^{PD} \end{array}\right.$$

where *i* indicates the subject and $F_{ij}$ is the value of feature *j* for subject *i*.

A generative model was trained to learn optimal weights for each labeling function, yielding probabilistic labels. A denoising neural network was subsequently employed to refine these labels, enhancing their accuracy and robustness. The design and architecture of the denoising network are detailed in Section S3 of the Supplementary Materials.

### 2.5. Cross-Validation Strategy

To evaluate the performance of the proposed approach, a participant-based leave-one-out cross-validation (LOOCV) strategy was employed [16]. In each iteration, features from the left and right hands of 19 HC and PD participants were used independently to generate the label matrix and train the denoising network. Thus, a training set of 36 samples and a test set of 2 samples were built. The features from both hands of the remaining participants served as the test set in each iteration.

### 2.6. Feature Fusion and Classification

**Figure 2:**
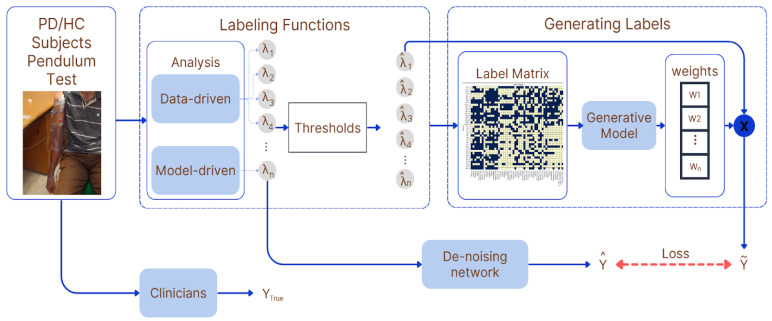
Overview of the proposed hybrid framework using weak supervision to fuse model-driven and data-driven features for classifying rigidity in PD. *Y_True_* represents ground-truth labels (e.g., UPDRS scores), while *Y* denotes predicted outputs from the integrated model.

Our hybrid framework combines model-driven and data-driven features into a unified classification model. The model-driven features provide a strong biomechanical foundation rooted in the physical dynamics of the pendulum motion. In contrast, the data-driven features capture statistical patterns and non-linear relationships in the sensor data. By integrating both types of features, the approach leverages the strengths of each: model-driven features offer interpretable, physically meaningful metrics, while data-driven features provide flexibility in capturing complex patterns specific to PD. An overview of the entire methodology is illustrated in Figure 2. Model-driven features (e.g., damping ratio) and data-driven features (e.g., wavelet coefficients) are extracted from IMU data, fused via weak supervision to generate probabilistic labels (*Y*), refined through a denoising network, and used for classification, comparing against *YTrue* for validation.

By using weak supervision to fuse these complementary perspectives, the model creates a probabilistic labeling framework that improves interpretability and performance. This approach is particularly beneficial given the variability in patient presentations and the limited availability of labeled clinical data.

## 3. Results

The results are organized into two main sections: (1) PD/HC Classification and (2) Rigidity Score Estimation. Also, evaluation of Weak Supervision is discussed in the Supplementary Materials Section S4. Each section compares the performance of data-driven, model-driven, and the integrated method, emphasizing the improvements obtained through the application of weak supervision.

### 3.1. PD/HC Classification

**Figure 3:**
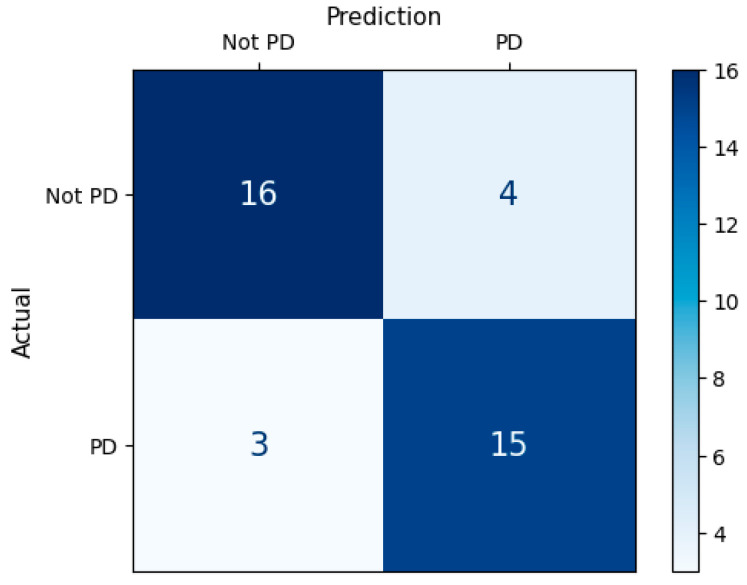
Confusion matrix of the integrated approach for classifying PD and HC participants.

We evaluated the classification performance of data-driven, model-driven, and integrated approaches for distinguishing individuals with Parkinson’s disease (PD) from healthy controls (HC). The performance metrics are summarized in Table 1, and the confusion matrix of the integrated approach is shown in Figure 3.

Model-Driven Approach: Leveraging biomechanical features such as damping ratio and decay rate, this approach achieved an improved accuracy of 0.71, a precision of 0.72, and an F1-score of 0.70.

Data-Driven Approach: This approach utilized statistical features derived from angular velocity signals. It achieved an accuracy of 0.66, a precision of 0.67, and an F1-score of 0.64. Detailed performance metrics of the data-driven approach are presented in Supplementary Section S1.

Integrated Approach with Weak Supervision: Compared to the data-driven method, the integrated approach improved performance to 0.71 across all three metrics, representing a 10% gain. This improvement stems from fusing biomechanical features with statistical descriptors, while weak supervision refines noisy or incomplete labels, enhancing robustness.

Relative to the model-driven method, which relies solely on biomechanical parameters such as damping ratio and decay rate, the integrated approach achieved nearly identical accuracy (0.71 vs. 0.71), precision (0.71 vs. 0.72), and F1-score (0.71 vs. 0.70). While the model-driven approach offers simplicity, it may be less resilient to the complex variability of PD motor patterns.

The hybrid framework, therefore, combines the strengths of both paradigms: biomechanical parameters provide physical interpretability, while statistical features (e.g., wavelet coefficients) capture non-linear dynamics. Weak supervision harmonizes these heterogeneous signals, reducing dependence on complete manual labels and supporting more generalizable, clinically interpretable classification.

Feature importance analysis revealed that the most predictive features were the maximum detail coefficient from the wavelet transform and the damping ratio. These findings support the hypothesis that combining physical and statistical descriptors captures complementary facets of PD-related motor dysfunction.

### 3.2. Rigidity Score Estimation

This analysis assessed the ability of each method to predict clinical rigidity scores for both the dominant and non-dominant arms.

Model-Driven Analysis: A strong correlation was found between the effective decay rate and clinical rigidity scores in PD subjects during their OFF-medication state (Pearson’s *r* = 0.78, *p* < 0.001).

Data-Driven Analysis: Machine learning models (Random Forest and Decision Tree) were used to predict rigidity scores (discrete values: 0, 1, 2) based on extracted features. The balanced accuracy reached 0.68 and improved further after the Synthetic Minority Oversampling Technique (SMOTE) was applied for class balancing (see Supplementary Section S1 for detailed metrics).

## 4. Discussion

This proof-of-concept study presented a novel framework for assessing shoulder rigidity in PD by combining model-driven and data-driven approaches, integrated through a weak supervision strategy. Our findings demonstrate the potential of this hybrid framework in a small cohort to provide a more objective, scalable, and interpretable assessment of rigidity, addressing key limitations of both traditional clinical methods and existing automated sensor-based approaches. However, generalizability remained limited due to the small sample size and potential risks of overfitting in machine learning models. Larger-scale validation is essential to confirm these findings.

### 4.1. Advantages of the Integrated Approach

The integration of model-driven and data-driven features using weak supervision provided several key advantages:

Model-driven features, such as damping ratios and decay rates, offer interpretable insights rooted in the physical dynamics of limb movement during the pendulum test. These parameters align well with clinical concepts of rigidity and are consistent with prior biomechanical assessments of neuromuscular impairment. In contrast, data-driven features, derived from statistical patterns in sensor data, capture complex, non-linear relationships that are not easily represented by physical models alone. This combination bridges the gap between biomechanics and machine learning, providing a comprehensive understanding of motor dysfunction in PD, in line with previous calls for hybrid modeling in clinical movement analysis.

Although the primary aim of this integration was not solely to enhance accuracy, the fusion of model- and data-driven features—unified via weak supervision—yielded a 10% increase in PD/HC classification accuracy compared to the data-driven method alone. The probabilistic labeling framework enabled effective learning from noisy and incomplete labels, reducing dependency on fully annotated datasets. This is particularly beneficial given the known variability in motor symptoms and disease progression across PD populations. These results support our initial hypothesis that hybrid integration would improve both generalization and robustness.

Beyond the 10% accuracy improvement over data-driven methods, the hybrid framework offers enhanced robustness by combining interpretable biomechanical insights (e.g., damping ratio) with flexible statistical patterns, potentially improving generalizability across PD subgroups with varying symptom profiles. It also captures complex motor patterns, such as transient disruptions, that single paradigms might miss, fostering better interpretability for clinical decision-making.

While the integrated and model-driven approaches achieved comparable accuracy, the hybrid model provides distinct advantages. Its fusion of statistical and biomechanical features strengthens resilience against sensor noise, broadens the detection of motor abnormalities (including non-linear dynamics captured by wavelet coefficients), and improves adaptability to diverse clinical contexts. Together, these benefits reinforce its utility for real-world PD assessment.

Traditional tools such as the UPDRS are inherently subjective, prone to inter-rater variability, and may overlook subtle changes in rigidity. Our framework offers a quantitative alternative by extracting sensor-derived features that were significantly correlated with clinical rigidity scores (*p* < 0.001). Among these, the effective decay rate emerged as a strong candidate biomarker for both diagnosis and monitoring, echoing findings from earlier pendulum-based assessments in clinical studies.

Compared to existing IMU-based data-driven studies, many of which focus on gait features, our hybrid framework strikes a better balance between performance and interpretability. For instance, [18] reported IMU-based PD gait classification accuracies ranging from 63% to 80%—dropping to 96% only for highly tuned ensemble methods—yet these remain largely black-box approaches [18]. Another study using deep learning achieved up to 92% classification accuracy using raw gait signals [19]. In contrast, our hybrid approach achieves an accuracy of 71%, which, while modest, offers interpretable biomechanical features (e.g., damping ratio) plus the robustness advantages of weak supervision—an important trade-off in clinically constrained pilot studies.

In comparison with broader wearable sensor applications, refs. [20] reviewed PD-related wearable systems and noted that most efforts (<15 patients) focused on body motion analysis, particularly gait, with limited integration of rigorous interpretability or hybrid modeling [20]. More recently, in ref. [21], they highlighted the need for wearable sensors to support real-world, scalable monitoring of Parkinsonian symptoms like tremor and rigidity—needs directly aligned with our hybrid framework’s design.

Our hybrid framework, therefore, offers clear advantages: It blends biomechanical insights with statistical pattern capture, enabling interpretability that purely data-driven methods often lack, and robustness that purely model-based approaches struggle to maintain. The use of weak supervision helps to mitigate data limitations and label noise—an important benefit not addressed by many existing methods. Furthermore, by facilitating remote, sensor-based monitoring of rigidity, our approach supports telemedicine and scalable clinical applications—competencies not supported by robotic or lab-bound systems. These strengths position our framework as an accessible, interpretable, and generalizable advance in PD rigidity assessment.

### 4.2. Role of Weak Supervision in Harmonizing Features

Weak supervision played a critical role in aligning model-driven and data-driven representations. Firstly, through the use of heuristic rules, generative models, and a denoising network, weak supervision enabled the generation of high-quality, probabilistic labels from partially labeled data. This significantly reduced the need for time-consuming manual annotation, a well-known barrier in medical research. Also, the denoising network refined the noisy outputs of initial labeling functions, improving confidence and mitigating the impact of outliers. This refinement enhanced both the robustness and precision of the final classification model, enabling more reliable detection of PD-specific motor signatures.

### 4.3. Refining Traditional Pendulum Tests with Novel Biomarkers

Our hybrid framework advances rigidity assessment in PD by refining the biomechanical principles of the Wartenberg pendulum test, which relies on features such as first swing excursion, relaxation index, maximum velocity, swing time, and plateau angle [4]. Feature importance analysis identified the damping ratio, decay rate, and maximum detail coefficient from wavelet transforms as highly predictive, surpassing traditional metrics like maximum velocity (Section 3.1). The damping ratio and decay rate, derived from a second-order linear time-invariant model, align closely with the Wartenberg test’s relaxation index and swing time, quantifying oscillation decay due to rigidity-induced resistance. Their strong correlation with UPDRS rigidity scores (*r* = 0.78, *p* < 0.001) validates their clinical utility as biomechanical biomarkers. The maximum detail coefficient, capturing high-frequency movement fluctuations, complements these by detecting PD-specific jerks, which are absent in traditional tests. By focusing on shoulder girdle rigidity—linked to decreased arm swing, an early PD sign—our sensor-based approach enables objective, remote monitoring, enhancing early diagnosis and telemedicine applications.

### 4.4. Re-Evaluating Rigidity’s Biomechanical Dynamics

A key finding is the prominence of the damping ratio and decay rate as biomarkers of PD rigidity, despite clinical teachings that rigidity is velocity-independent, unlike spasticity [2]. In a spring-damper model, the damping ratio depends on the velocity-proportional damping coefficient (*C*), which may reflect a biomechanical modeling artifact suggesting potential velocity-related influences in rigidity quantification (Supplementary Section S1). This interpretation challenges conventional understanding and warrants further investigation as a hypothesis, as these features, along with the maximum detail coefficient, capture dynamic resistance and transient disruptions in passive arm swing, isolated from bradykinesia via the modified pendulum test (Section 2.2). Their predictive power (Section 3.1) and alignment with Wartenberg test metrics (e.g., relaxation index, swing time) position them as novel biomechanical biomarkers, offering greater precision than traditional features like maximum velocity. This insight, enabled by wearable sensors and weak supervision, supports scalable, objective rigidity assessment, paving the way for refined diagnostic criteria and longitudinal monitoring in PD management.

### 4.5. Limitations and Future Work

While promising, our approach has several limitations: (1) The relatively small cohort (19 subjects) may limit the generalizability of our findings. Specific plans include multi-center collaborations to recruit at least 30 participants across Hoehn and Yahr stages 1–4, incorporating diverse demographics (e.g., age, ethnicity, comorbidities) to validate the framework’s performance, and enhance robustness and generalizability, building on this pilot as a foundation for scalable clinical applications. This will build on our preliminary findings and address overfitting risks in the current small cohort. (2) Although sensor placement was standardized, slight variations could introduce signal differences affecting feature extraction. Future work should assess the sensitivity of the framework to sensor configuration and develop calibration procedures to ensure robustness. (3) While weak supervision reduces overfitting risk by leveraging diverse weak signals, the added complexity from integrating two modeling paradigms may still pose a risk with limited data. To mitigate this, advanced regularization, dimensionality reduction, or feature selection techniques may be explored.

### 4.6. Clinical Implications and Future Directions

The framework presented here holds significant potential for improving PD care. Firstly, the use of lightweight, wearable sensors combined with automated analysis enables remote monitoring of rigidity, making the method well-suited for telemedicine, especially important in the post-COVID era and underserved or rural populations. Secondly, by detecting subtle changes in rigidity that might be missed by clinical observation, our method may facilitate earlier diagnosis and enable more precise tracking of disease progression and treatment response.

Future research will focus on expanding the dataset through multi-center collaborations, incorporating additional modalities such as force or tactile sensors, and extending the framework via transfer learning to other movement disorders like atypical Parkinsonism. These steps will enhance the generalizability and clinical utility of our approach.

## 5. Conclusions

This study introduces a novel, weakly supervised hybrid framework for assessing shoulder rigidity in Parkinson’s Disease by integrating model- and data-driven features. Our results demonstrate that this approach enhances classification performance and interpretability and provides a scalable, objective clinical and remote monitoring tool. By addressing the limitations of both traditional clinical assessments and sensor-based machine learning models, this framework lays the foundation for more accurate, efficient, and personalized care in PD management.

## Figures and Tables

**Table 1:** Performance metrics for classifying PD and HC subjects using data-driven, model-driven, and integrated approaches.

Method	Accuracy	Precision	F1-Score
Integrated Approach (Ours)	0.71	0.71	0.71
Data-Driven Approach (Ours)	0.66	0.67	0.64
Model-Driven Approach (Ours)	0.71	0.72	0.70
Baseline (RF, Leave-One-Out)	0.73	0.36	0.34
Baseline (DT, Leave-One-Out)	0.60	0.31	0.23
Baseline (RF, 5-Fold Cross Validation)	0.70	0.75	0.60

## Data Availability

The datasets presented in this article are not readily available because they contain sensitive clinical information and are subject to institutional ethics restrictions, even though the study has been completed. Requests to access the datasets should be directed to Dr. Martin J. McKeown (martin.mckeown@ubc.ca).

## Supplementary Materials

The following supporting information can be downloaded at: https://www.mdpi.com/article/10.3390/s25196019/s1, Table S1: The complete list of all extracted features: Model-driven and data-driven. Table S2: Detailed characteristics of PD subjects. For rigidity, ‘LH’ denotes left hand, ‘RH’ denotes right hand, ‘LL’ denotes left leg, ‘RL’ denotes right leg, and ‘N’ denotes neck. Figure S1: A scheme of a pendulum. CG, center of gravity; L, length; r, radius. Figure S2: A sample angular velocity of the arm swing (raw output). Figure S3: The correlation of effective decay rate and rigidity. Figure S4: Correlation between labelling functions, weak labels (Ỹ), refined labels (Ŷ), and true labels. The denoising model improves accuracy by correcting label noise. Figure S5: Network graph showing correlations between labelling functions. Only correlations above 0.7 are shown. Nodes represent individual functions; edges indicate shared information. Figure S6: Graphical model of correlations between labelling functions as well as the Label Model. Only correlation values of more than 0.7 are presented.

## Abbreviations

The following abbreviations are used in this manuscript:

PD	Parkinson’s Disease
UPDRS	Unified Parkinson’s Disease Rating Scale
LTI	Linear Time Invariant

## References

[1] Dorsey E.R., Sherer T., Okun M.S., Bloem B.R. (2018). The Emerging Evidence of the Parkinson Pandemic. J. Park. Dis..

[2] Postuma R.B., Berg D., Stern M., Poewe W., Olanow C.W., Oertel W., Obeso J., Marek K., Litvan I., Lang A.E. (2015). MDS clinical diagnostic criteria for Parkinson’s disease. Mov. Disord..

[3] Ferreira-Sánchez M.R., Moreno-Verdú M., Cano-de-la-Cuerda R. (2020). Quantitative Measurement of Rigidity in Parkinson’s Disease: A Systematic Review. Sensors.

[4] Martino G., McKay J.L., Factor S.A., Ting L.H. (2020). Neuromechanical Assessment of Activated vs. Resting Leg Rigidity Using the Pendulum Test Is Associated with a Fall History in People with Parkinson’s Disease. Front. Hum. Neurosci..

[5] Werning A., Umbarila D., Fite M., Fergus S., Zhang J., Molnar G.F., Johnson L.A., Wang J., Vitek J.L., Escobar Sanabria D. (2022). Quantifying Viscous Damping and Stiffness in Parkinsonism Using Data-Driven Model Estimation and Admittance Control. J. Med. Devices.

[6] Lu R., Xu Y., Li X., Fan Y., Zeng W., Tan Y., Ren K., Chen W., Cao X. (2020). Evaluation of Wearable Sensor Devices in Parkinson’s Disease: A Review of Current Status and Future Prospects. Park. Dis..

[7] Karniadakis G.E., Kevrekidis I.G., Lu L., Perdikaris P., Wang S., Yang L. (2021). Physics-informed machine learning. Nat. Rev. Phys..

[8] Ratner A., Bach S.H., Ehrenberg H., Fries J., Wu S., Ré C. (2017). Snorkel: Rapid training data creation with weak supervision. Proc. VLDB Endow..

[9] Zhang A., Cebulla A., Panev S., Hodgins J., De la Torre F. (2017). Weakly-supervised learning for Parkinson’s Disease tremor detection. Proceedings of the 2017 39th Annual International Conference of the IEEE Engineering in Medicine and Biology Society (EMBC).

[10] Yue P., Li Z., Zhou M., Wang X., Yang P. (2024). Wearable-Sensor-Based Weakly Supervised Parkinson’s Disease Assessment with Data Augmentation. Sensors.

[11] Djurić-Jovicić M.D., Jovicić N.S., Radovanović S.M., Stanković I.D., Popović M.B., Kostić V.S. (2014). Automatic identification and classification of freezing of gait episodes in Parkinson’s disease patients. IEEE Trans. Neural Syst. Rehabil. Eng. A Publ. IEEE Eng. Med. Biol. Soc..

[12] Ashoori A. (2014). Analysis of Motor Performance in Parkinson’s Disease Through LTI Dynamical Systems.

[13] Cooper P. (2005). DeJong’s The Neurologic Examination. 2005. Sixth edition. By William W. Campbell. Published by Lippincott, Williams & Wilkins. 671 pages. C$140 approx. Can. J. Neurol. Sci. J. Can. Des Sci. Neurol..

[14] Patera M., Zampogna A., Pietrosanti L., Asci F., Falletti M., Pinola G., Bianchini E., Di Lazzaro G., Rosati V., Grillo P. (2025). Abnormal arm swing movements in Parkinson’s disease: Onset, progression and response to L-Dopa. J. Neuroeng. Rehabil..

[15] Huang X., Mahoney J.M., Lewis M.M., Du G., Piazza S.J., Cusumano J.P. (2012). Both coordination and symmetry of arm swing are reduced in Parkinson’s disease. Gait Posture.

[16] Moore S.T., MacDougall H.G., Ondo W.G. (2008). Ambulatory monitoring of freezing of gait in Parkinson’s disease. J. Neurosci. Methods.

[17] Gourrame K., Griškevičius J., Haritopoulos M., Lukšys D., Jatužis D., Kaladytė-Lokominienė R., Bunevičiūtė R., Mickutė G. (2023). Parkinson’s disease classification with CWNN: Using wavelet transformations and IMU data fusion for improved accuracy. Technol. Health Care Off. J. Eur. Soc. Eng. Med..

[18] Caramia C., Torricelli D., Schmid M., Munoz-Gonzalez A., Gonzalez-Vargas J., Grandas F., Pons J.L. (2008). IMU-Based Classification of Parkinson’s Disease from Gait: A Sensitivity Analysis on Sensor Location and Feature Selection. IEEE J. Biomed. Health Inform..

[19] Carvajal-Castaño H.A., Pérez-Toro P.A., Orozco-Arroyave J.R. (2022). Classification of Parkinson’s Disease Patients—A Deep Learning Strategy. Electronics.

[20] Rovini E., Maremmani C., Cavallo F. (2017). How Wearable Sensors Can Support Parkinson’s Disease Diagnosis and Treatment: A Systematic Review. Front. Neurosci..

[21] Moreau C., Rouaud T., Grabli D., Benatru I., Remy P., Marques A.-R., Drapier S., Mariani L.-L., Roze E., Devos D. (2023). Overview on wearable sensors for the management of Parkinson’s disease. Npj Park. Dis..

