# Supplementary material for: Automated Shoulder Girdle Rigidity Assessment in Parkinson’s Disease via an Integrated Model- and Data-Driven Approach
Source: Sensors (Basel). 2025 Oct 1;25(19):6019. doi: 10.3390/s25196019 (PMC12526519; doi:10.3390/s25196019)
Supplement: Supplementary file 1 [file sensors-25-06019-s001.zip › sensors-3809265-supplementary.pdf]

Supplementary materials

Section S1: Details of the Data-Driven Method

Using a data-driven approach, we discovered that features from the wrist gyroscope sensor, particularly in the *z*-axis, contain valuable information for distinguishing between Parkinson's Disease (PD) and healthy controls (HC). In our analysis, we applied 5-fold cross-validation with Decision Trees (DT) and Random Forest (RF) classifiers, achieving accuracies of 0.686 (σ: 0.153, F1: 0.77) and 0.761 (σ: 0.134, F1: 0.72), respectively.

Notably, the maximum detail coefficient derived from the Wavelet Transform of the angular velocity signal emerged as the most important feature according to the RF feature importance analysis. This finding supports our hypothesis that the maximum detail coefficient, a characteristic in the time–frequency domain, plays a crucial role in differentiating PD from HC during the pendulum test. The pendulum test inherently involves changes in both time and frequency domains, and the rigidity associated with PD impacts both aspects.

Through our data-driven approach, we discovered that features extracted from the wrist gyroscope sensor's *x*-axis contain significant information relevant to estimating arm rigidity scores. This finding is particularly intriguing, as the *x*-axis does not align with the direction of the arm swing, while previous model-driven methods highlighted only the z-axis as promising.

To address the class imbalance in hand rigidity score estimation (scores 0, 1, 2), we adopted the Synthetic Minority Over-Sampling Technique (SMOTE) within a participant-level leave-one-out cross-validation (LOOCV) framework. This protocol was explicitly designed to prevent data leakage and ensure unbiased performance estimates. For each LOOCV fold, the following steps were performed:

Data Splitting: The dataset was divided at the participant level, with data from 18 participants (36 hand samples) forming the training set and data from the held-out participant (2 hand samples) forming the test set.
In-Fold SMOTE Application: SMOTE was applied exclusively to the training set to balance class priors across rigidity scores (0, 1, 2). Synthetic samples were generated using only training features and labels to ensure independence from the test set.
Model Training: Decision Tree (DT) and Random Forest (RF) classifiers were trained on the SMOTE-augmented training set.
Model Evaluation: The trained model was evaluated on the untouched test set from the held-out participant, with no synthetic samples or resampling applied.

Iteration Across Folds: This process was repeated for all 19 folds to obtain participant-independent performance metrics.
Mean classification accuracies (± standard deviation) across folds were as follows:
Without SMOTE: DT = 0.66 ± 0.23, RF = 0.68 ± 0.18
With In-Fold SMOTE: DT = 0.92 ± 0.10, RF = 0.86 ± 0.09

Feature importance analyses identified the following discriminative features:
x-axis: Area under the curve (AUC) and maximum approximation coefficients of the angular velocity signal (deg/s).
z-axis: Area under the curve (AUC) and maximum frequency of the angular velocity signal.
These findings confirm the complementary role of features extracted from different sensor axes in capturing rigidity-related patterns.

Table S1: The complete list of all extracted features: Model-driven and data-driven

Feature Name	Definition	Why Useful for Rigidity Assessment?
**Damping Ratio**	Quantifies how oscillations in the arm swing decay after perturbation; estimated from a second-order LTI model.	Reflects mechanical damping associated with rigidity; higher damping ratios indicate stiffer movement, enabling discrimination between PD and healthy controls.
**Decay Rate**	The rate at which arm swing oscillations diminish over time.	Provides a dynamic measure of how quickly movement subsides, directly correlating with rigidity severity and offering biomechanical interpretability.
**Natural Frequency (Wn)**	The frequency at which the arm would oscillate without damping.	Captures intrinsic limb properties (mass, inertia); differences may reveal compensatory changes in movement strategy in PD vs. controls.
**Damped Natural Frequency (Wd)**	The oscillation frequency including damping effects.	Integrates stiffness and damping into a single dynamic feature; informative for modeling altered arm swing in PD.
**Area Under the Curve (AUC)**	Integral of angular velocity over time.	Measures overall movement magnitude; reduced AUC often reflects bradykinesia and reduced swing amplitude in PD.
**Mean Angular Velocity**	Average angular velocity during arm swing.	Indicates general movement speed; lower values are characteristic of PD motor slowing.
**Standard Deviation (SD) of Angular Velocity**	Dispersion of angular velocity values.	Captures variability in movement execution, relevant as PD often leads to reduced or erratic variability.
**Variance of Angular Velocity**	The squared dispersion around the mean angular velocity.	Another measure of variability; can enhance model robustness by capturing subtle differences in movement consistency.
**Peak Angular Velocity**	Maximum angular velocity reached during arm swing.	Reflects maximal movement vigor; typically reduced in PD, supporting discrimination from controls.

**Dominant Frequency (FFT)**	Principal frequency component extracted via FFT.	Captures the rhythmicity of swing; altered frequency content is linked to motor impairment in PD.
**Wavelet Frequency Features**	Additional frequency domain features from wavelet decomposition capturing transient changes.	Sensitive to non-stationary disruptions and micro-freezing episodes in PD swing patterns.
**Wavelet Max Detail Coefficient**	Maximum detail coefficient from continuous wavelet transform (CWT), reflecting high-frequency temporal-spectral components.	Highlights sudden movement fluctuations and jerks, often more prevalent in PD rigidity.
**Wavelet Max Approximation Coefficient**	Maximum approximation coefficient from CWT, representing low-frequency components and overall swing trends.	Captures sustained movement characteristics and underlying periodicity changes associated with rigidity and bradykinesia.

Section S2: Details of the Data Collection

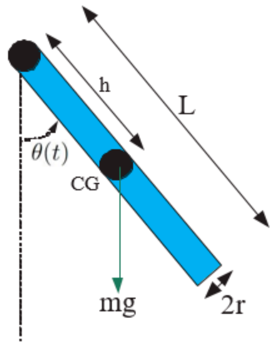

Figure S1. A scheme of a pendulum. CG, center of gravity; L, length; r, radius.

Table S2: Detailed characteristics of PD subjects. For rigidity, 'LH' denotes left hand, 'RH' denotes right hand, 'LL' denotes left leg, 'RL' denotes right leg, and 'N' denotes neck.

**Subj. No.**	Gender	Handed	Mass (lb)	Arm length	Arm radius	UPDRS	Rigidity
1	M	R	175	22.5″	1.45″	18	0,0,0,1,1
2	F	R	120	20″	1.48″	22	0,1,0,1,0
3	M	L	197	22″	1.75″	21	1,2,1,2,2
4	F	R	130	22″	1.32″	32	2,1,2,2,2
5	M	R	162	20.5″	1.48″	35	2,2,2,2,2
6	F	R	175	19.5″	1.42″	32	1,1,1,2,2
7	M	R	165	20.5″	1.34″	22	1,1,1,1,1
8	M	R	190	23″	1.67″	24	1,1,1,1,2
9	M	R	155	21.5″	1.53″	21	1,1,1,1,1
10	M	R	166	22.5″	1.57″	10	1,1,0,1,0

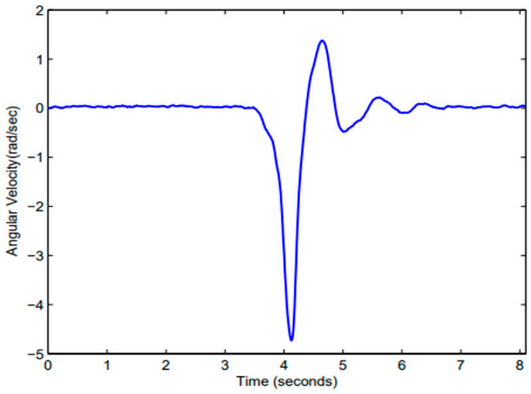

Figure S2. A sample angular velocity of the arm swing (raw output)

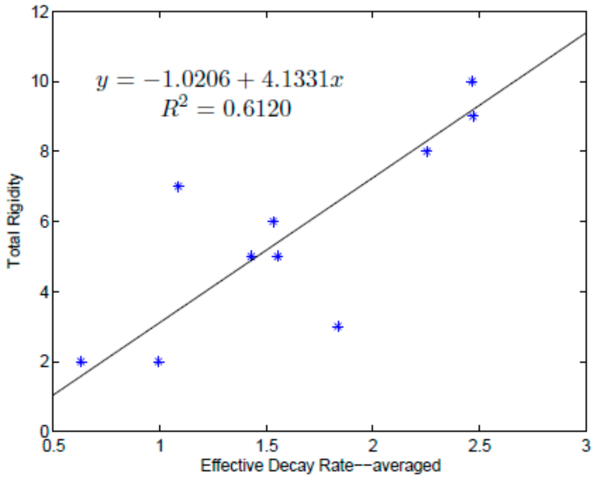

Figure S3. The correlation of effective decay rate and rigidity.

Section S3: Details of the Network

Architecture details

Following the training of the label model, the learned weights corresponding to each feature (51 features in total) are extracted and normalized. These weights are then multiplied with the initial labeled features to generate the probabilistic labels ($\tilde{Y}$ illustrated in Fig. 1). The denoising network is to directly learn the outputs from comprehensive features of participants' hand motion by fine-tuning thresholds to be more accurate than the initial thresholds. The network takes as input the motion-derived features, encompassing both data-driven and model-driven approaches. Prior to input, all features are standardized by subtracting their mean and dividing by their standard deviation to ensure consistency and improve training stability. The network utilizes a noise-aware loss function, which is based on the discrepancy between the output of the denoising network and labels generated by the generative model.

The denoising network is designed as a feedforward neural network with three layers. The architecture consists of:

- Input Layer: 51 neurons corresponding to the features.
- Hidden Layer: 120 neurons with a ReLU activation function.
- Output Layer: A single neuron with a sigmoid activation function to produce a probabilistic output.

Training

The whole pipeline was trained and evaluated using participant-based leave-one-out cross-validation (LOOCV) approach
. Thus, we used 18 out of 19 subjects for training, leaving a subject out every time. Therefore, we completed a total of 19 training/validation experiments.
The network is trained using the binary cross-entropy loss function with the Adam optimizer to ensure effective weight updates. The training process is conducted over 50 epochs, with a batch size of one, meaning that the movement data for each hand of each subject is individually used to update the network weights. To limit overfitting, the denoising network was kept minimal, with a single hidden layer. Though no explicit regularization was applied, LOOCV and input standardization provided effective control in this low-data setting.

Section S4: Evaluation of Weak Supervision

The effectiveness of the weak supervision pipeline was assessed by comparing the initial labels generated by heuristic functions (Label Model output, $\hat{Y}$) with the refined labels produced by the denoising network ($\hat{Y}$), as shown in **Figure S4**. The strong alignment between the refined labels ($\hat{Y}$) and true labels indicates that the denoising network effectively corrects noise in the initial weak labels. This is critical in clinical settings where labelled data are often sparse or noisy, ensuring that the model's predictions are more reliable for PD diagnosis and rigidity assessment.

Label Refinement: The denoising network substantially improved label quality by correcting noisy predictions. Correlation analyses confirmed strong alignment between refined and true labels.

Label Matrix Visualization: As shown in **Figure S5**, the label matrix offers a detailed view of feature contributions per subject. Features are sorted by their discriminatory power. The final column shows the weighted aggregate per subject from the generative model. The graph also reveals synergistic relationships where labelling functions complement each other, enhancing the robustness of the label generation process. This synergy is crucial for capturing diverse aspects of rigidity, such as biomechanical and statistical patterns, in a unified model.

Function Correlation Graph: **Figure S6** presents a network graph depicting interrelationships among labelling functions. Edges indicate correlations greater than 0.7. This visualization helps identify redundancy and synergy among heuristics. The graphical

model provides a clear view of how individual labelling functions influence the final labels, enhancing the interpretability of the weak supervision process. This transparency is valuable in clinical applications, where understanding the basis of model decisions is critical for trust and adoption.

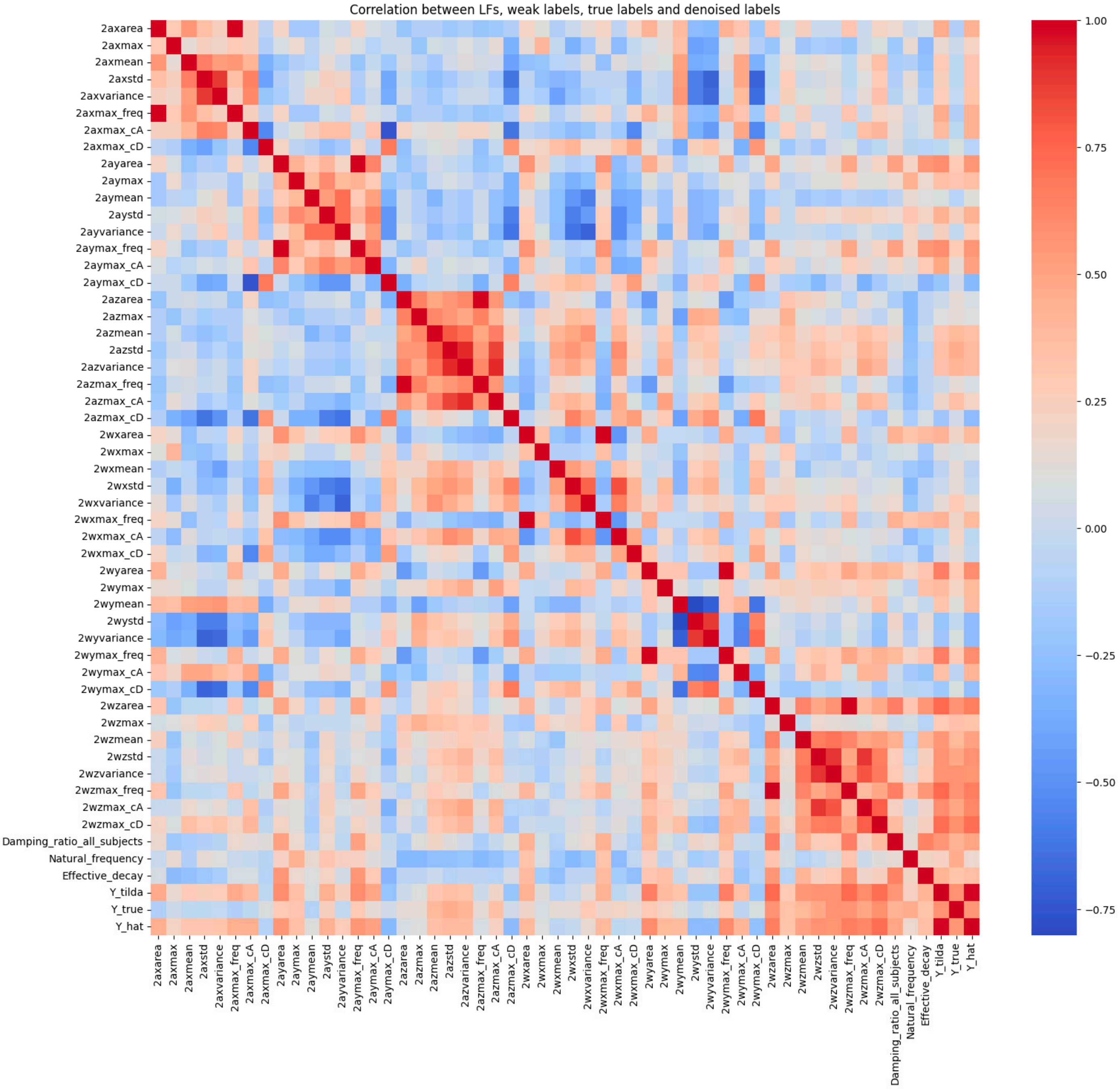

Figure S4. Correlation between labelling functions, weak labels ($\tilde{Y}$), refined labels ($\hat{Y}$), and true labels. The denoising model improves accuracy by correcting label noise.

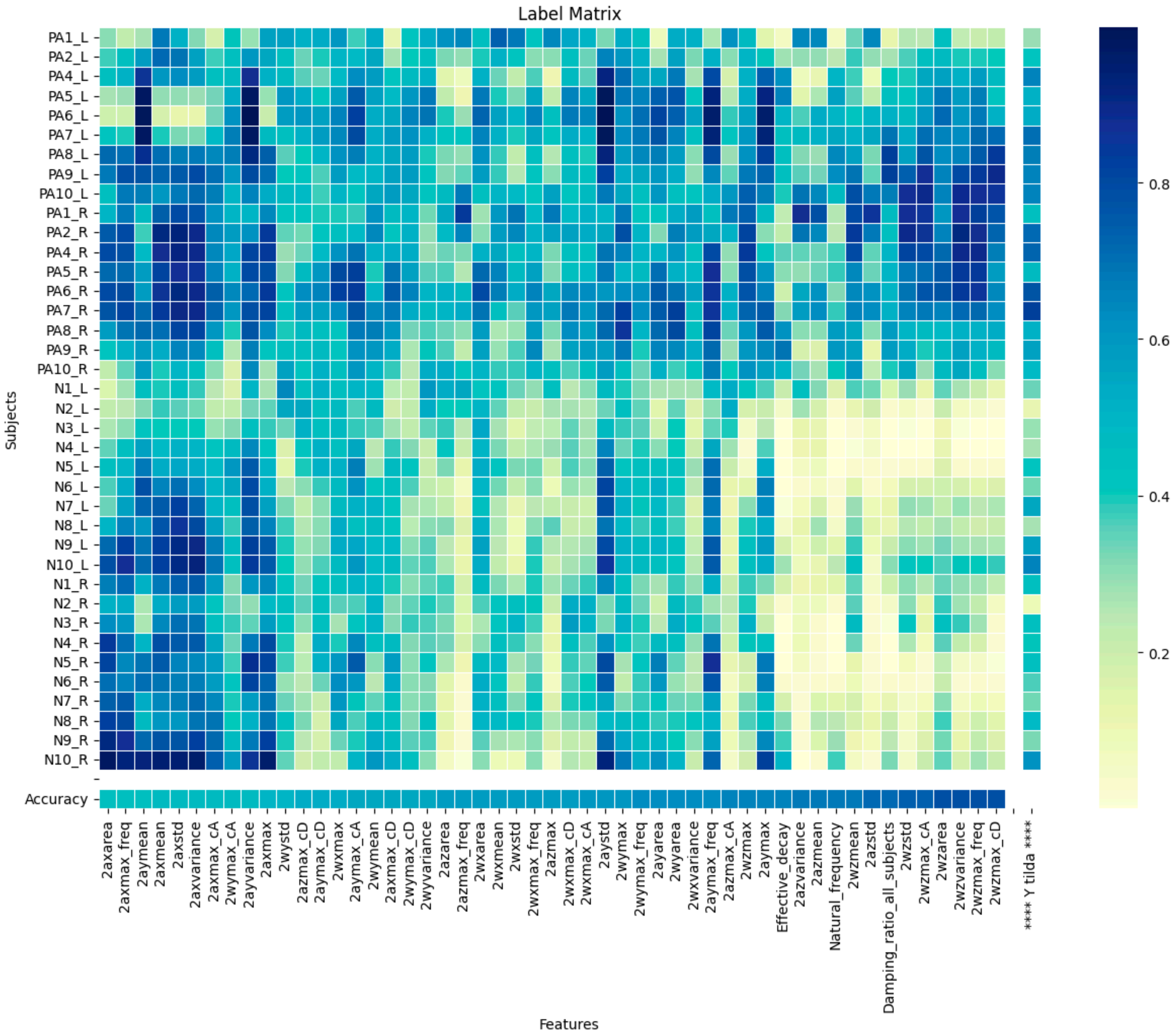

Figure S5. Network graph showing correlations between labelling functions. Only correlations above 0.7 are shown. Nodes represent individual functions; edges indicate shared information.

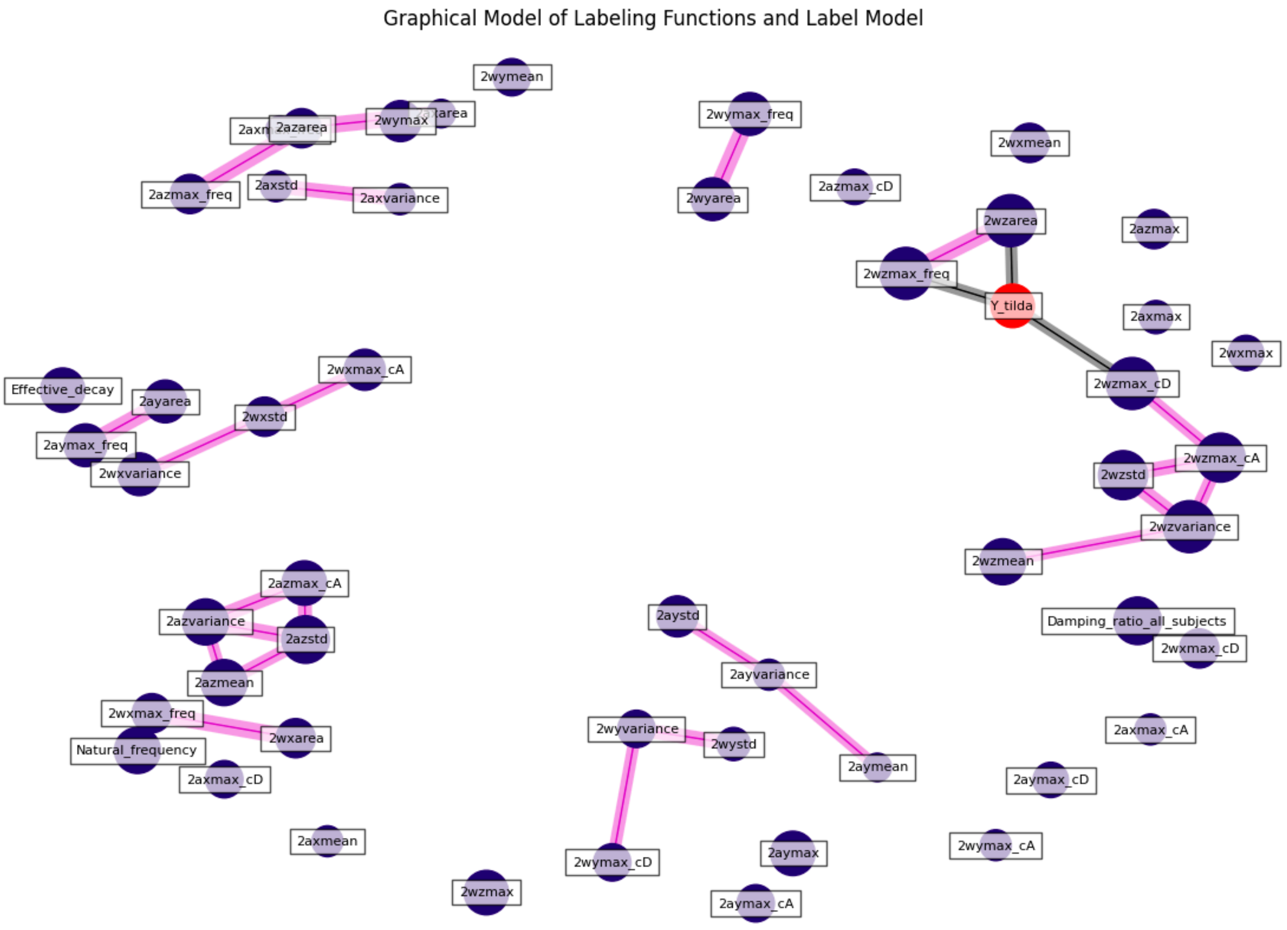

Figure S6. Graphical model of correlations between labelling functions as well as the Label Model. Only correlation values of more than 0.7 are presented.
